# Natriuretic peptide receptor guanylyl cyclase-A pathway counteracts glomerular injury evoked by aldosterone through p38 mitogen-activated protein kinase inhibition

**DOI:** 10.1038/srep46624

**Published:** 2017-04-21

**Authors:** Yukiko Kato, Kiyoshi Mori, Masato Kasahara, Keisuke Osaki, Akira Ishii, Keita P. Mori, Naohiro Toda, Shoko Ohno, Takashige Kuwabara, Takeshi Tokudome, Ichiro Kishimoto, Moin A. Saleem, Taiji Matsusaka, Kazuwa Nakao, Masashi Mukoyama, Motoko Yanagita, Hideki Yokoi

**Affiliations:** 1 Department of Nephrology, Graduate School of Medicine, Kyoto University, Kyoto, Japan; 2 School of Pharmaceutical Sciences, University of Shizuoka, Shizuoka, Japan; 3 Department of Nephrology and Kidney Research, Shizuoka General Hospital, Shizuoka, Japan; 4 Institute for Clinical and Translational Science, Nara Medical University Hospital, Kashihara, Japan; 5 Department of Nephrology, Kumamoto University Graduate School of Medical Sciences, Kumamoto, Japan; 6 Department of Biochemistry, National Cerebral and Cardiovascular Research Institute, Osaka, Japan; 7 Department of Endocrinology and Diabetes, Toyooka Public Hospital, Toyooka, Japan; 8 Children’s Renal Unit, Bristol Royal Hospital for Children, University of Bristol, Bristol, UK; 9 Department of Molecular Life Sciences, Tokai University School of Medicine, Isehara, Japan; 10 Medical Innovation Center, Graduate School of Medicine, Kyoto University, Kyoto, Japan

## Abstract

Guanylyl cyclase-A (GC-A) signaling, a natriuretic peptide receptor, exerts renoprotective effects by stimulating natriuresis and reducing blood pressure. Previously we demonstrated massive albuminuria with hypertension in uninephrectomized, aldosterone-infused, and high salt-fed (ALDO) systemic GC-A KO mice with enhanced phosphorylation of p38 mitogen-activated protein kinase (MAPK) in podocytes. In the present study, we examined the interaction between p38 MAPK and GC-A signaling. The administration of FR167653, p38 MAPK inhibitor, reduced systolic blood pressure (SBP), urinary albumin excretion, segmental sclerosis, podocyte injury, and apoptosis. To further investigate the local action of natriuretic peptide and p38 MAPK in podocytes, we generated podocyte-specific (pod) GC-A conditional KO (cKO) mice. ALDO pod GC-A cKO mice demonstrated increased urinary albumin excretion with marked mesangial expansion, podocyte injury and apoptosis, but without blood pressure elevation. FR167653 also suppressed urinary albumin excretion without reducing SBP. Finally, we revealed that atrial natriuretic peptide increased phosphorylation of MAPK phosphatase-1 (MKP-1) concomitant with inhibited phosphorylation of p38 MAPK in response to MAPK kinase 3 activation, thereby resulting in decreased mRNA expression of the apoptosis-related gene, *Bax*, and *Bax/Bcl2* ratio in cultured podocytes. These results indicate that natriuretic peptide exerts a renoprotective effect via inhibiting phosphorylation of p38 MAPK in podocytes.

Aldosterone has attracted much attention in the process of renal injury leading to proteinuria and chronic renal failure^1^. The mechanism underlying the contribution of aldosterone to worsening kidney function is thought to partly due to involve the activation of the mineralocorticoid receptor in podocytes, resulting in podocyte injury^2^, reactivation of renin-angiotensin-aldosterone system through positive feedback^3^, and the induction of reactive oxygen species (ROS) and activation of mitogen-activated protein kinases (MAPKs) in the kidney^4^. The site of action of aldosterone remains obscure, as aldosterone has been reported to exert its effect on podocytes^5^, mesangial cells^6^, endothelial cells^7^, and tubular cells^8^. In addition, aldosterone elevates blood pressure and regulates intraglomerular pressure^8^, which both contributes to the complex pathophysiology in aldosterone-induced renal injury.

Natriuretic peptides consist of atrial natriuretic peptide (ANP), brain natriuretic peptide (BNP), and C-type natriuretic peptide (CNP). ANP and BNP exert physiological effects such as diuresis and vasodilation through guanylyl cyclase-A (GC-A)/natriuretic peptide receptor-A (NPR-A)^9^. We previously demonstrated massive albuminuria, mesangial expansion, segmental sclerosis, and podocyte-injury with the elevation of systolic blood pressure in uninephrectomized, aldosterone-infused, and high salt diet-fed (ALDO) systemic GC-A knockout (KO) mice^10^. In the previous model, reduced blood pressure with the use of hydralazine fails to ameliorate renal injury; however, blockade of the renin angiotensin system by an angiotensin receptor blocker (ARB) or antioxidant tempol reduces glomerular injury^10^. Although our previous report may indicate that natriuretic peptides protect podocytes from aldosterone-induced renal injury, the precise site of action of natriuretic peptides/GC-A system, and the mechanism underlying amelioration of aldosterone-induced glomerular injury by natriuretic peptides have yet to be elucidated. In our previous study, we also demonstrated prominent phosphorylation of p38 MAPK in podocyte of ALDO GC-A KO mice^10^. Activation of p38 MAPK has been shown to be stimulated by UV light, heat, inflammation, and growth factors^11^. We and others have demonstrated that phosphorylation of p38 MAPK is activated in clinical glomerulonephritis, as well as in experimental podocyte injury glomerulopathy^10^,^12^,^13^. Blockade of p38 MAPK pathway reportedly ameliorates adriamycin nephropathy^12^ and crescentic glomerulonephritis^14^ in mice. However, the precise role of p38 MAPK in podocyte injury has yet to be elucidated. In addition, the mechanism underlying the renoprotective action of natriuretic peptides on podocytes remains to be clarified.

In the present study, we generated podocyte-specific (pod) GC-A conditional KO (cKO) mice and then examined the effects of p38 MAPK in systemic and pod GC-A cKO mice administered aldosterone, uninephrectomy, and a high salt diet.

## Results

### Inhibition of p38 MAPK ameliorates glomerular injury in ALDO systemic GC-A KO mice

**Figure 1:**
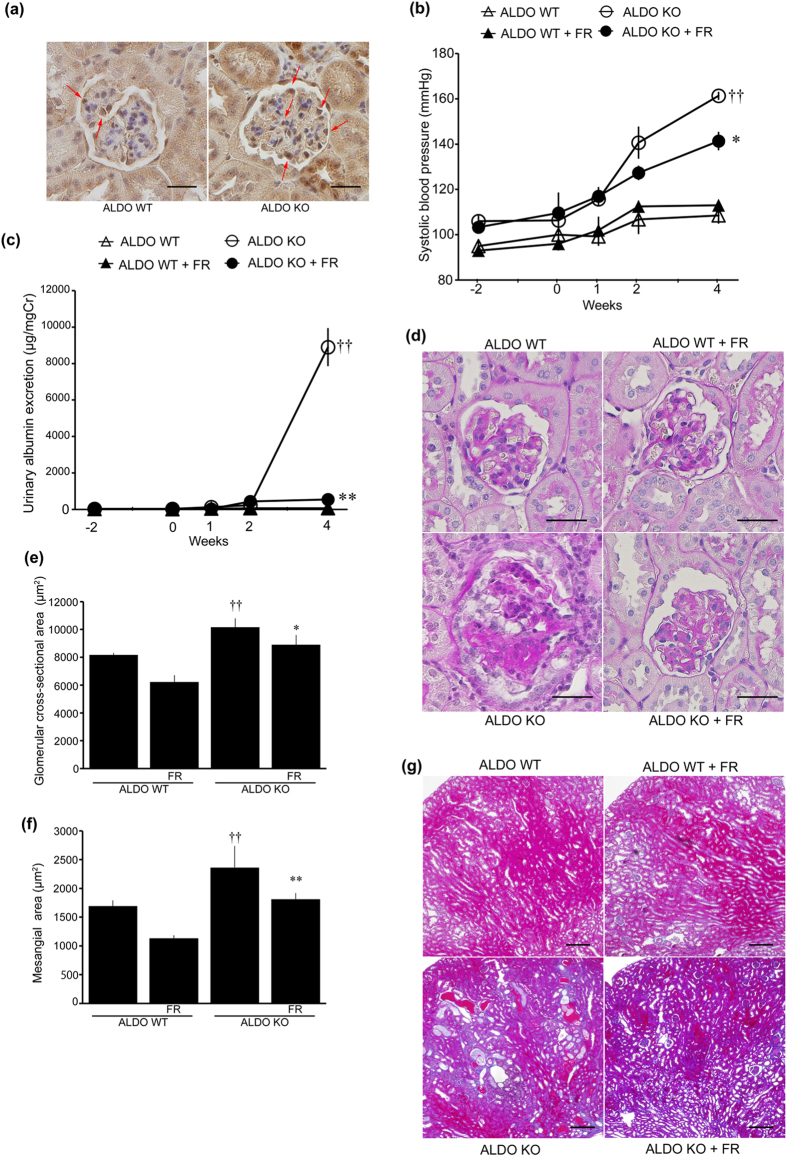
Inhibition of phospho-p38 MAPK in ALDO systemic GC-A KO mice. (**a**) Immunohistochemical study for phopho-p38 MAPK in glomeruli. Left panel shows a glomerulus in uninephrectomized, aldosterone-infused and high salt diet-fed (ALDO) wild-type mice. Right panel shows a glomerulus in ALDO systemic GC-A KO mice. Arrows, phospho-p38 MAPK-positive cells. Scale bar, 50 μm. (**b**) Time course of systolic blood pressure in ALDO wild-type mice, ALDO wild-type mice with FR167653, ALDO systemic GC-A KO mice, and ALDO systemic GC-A KO mice with FR167653. (**c**) Time course of albuminuria in these four groups. (**d**) Light microscopic analyses were performed at 4 weeks after aldosterone administration, stained with periodic acid-Schiff. In juxtamedullary glomeruli, ALDO systemic GC-A KO mice showed segmental sclerosis with glomerular hypertrophy. Treatment with FR167653 improved these changes. Scale bar, 50 μm. (**e**) Glomerular cross-sectional area and (**f**) mesangial area in juxtamedullary glomeruli at 4 weeks. (**g**) Histological examination of tubules and interstitial fibrosis by Masson’s trichrome-staining at 4 weeks. ALDO systemic GC-A KO mice exhibited tubular dilatation with protein cast deposition. These change were ameliorated by FR167653. Scale bar, 50 μm. n = 5, each. Mean ± SEM. ^††^*p* < 0.01 vs. ALDO wild-type mice. *p < 0.05, **p < 0.01 vs. ALDO systemic KO mice. WT, wild-type mice; KO, systemic GC-A knockout mice.

All ALDO mice were uninephrectomized and administered aldosterone and a 6% high salt diet for four weeks. First, we examined p38 MAPK phosphorylation in ALDO wild-type and systemic GC-A KO mice (Fig. 1a). Phosphorylation of p38 MAPK was predominantly detected in podocytes in ALDO wild-type mice (Fig. 1a). ALDO systemic GC-A KO mice demonstrated pronounced phosphorylation of p38 MAPK in podocytes (Fig. 1a), and had elevated systolic blood pressure (SBP) (Fig. 1b). Blockade of the p38 MAPK pathway using FR167653 (a p38 MAPK inhibitor) reduced SBP by 20 mmHg in ALDO systemic GC-A KO mice (Fig. 1b). Urinary albumin excretion in ALDO systemic GC-A KO mice was as high as 9,000 μg/mgCr; however, administration of FR167653 reduced urinary albumin excretion by 95% (Fig. 1c, Table 1). Body and kidney weights, and blood and urine parameters are presented in Table 1. ALDO systemic GC-A KO mice were found to have renal hypertrophy, and no effect of FR167653 on renal hypertrophy was observed. No significant differences in serum potassium, sodium, urinary potassium, or urinary sodium levels were observed (Table 1).

We examined renal histology at four weeks after aldosterone infusion. A mild mesangial expansion in superficial glomeruli was observed in ALDO wild-type mice (Supplementary Fig. S1a). In contrast, ALDO systemic GC-A KO mice had marked mesangial expansion with glomerular hypertrophy (Supplementary Fig. S1a–c). Administration of FR167653 significantly reduced mesangial expansion and glomerular hypertrophy (Supplementary Fig. S1a–c). In juxtamedullary glomeruli, ALDO systemic GC-A KO mice exhibited severe segmental sclerosis with glomerular hypertrophy (Fig. 1d). Treatment with FR167653 inhibited segmental sclerosis and glomerular hypertrophy in ALDO systemic GC-A KO mice (Fig. 1d–f). We further examined renal fibrotic changes. ALDO systemic GC-A KO mice exhibited tubular dilatation with protein cast deposition, and these changes were ameliorated by FR167653 (Fig. 1g).

Next, we examined gene expression in glomeruli. TGF-β1 (*Tgfb1*) is a profibrotic growth factor and is closely associated with matrix accumulation and renal fibrosis. Analysis of glomerular expression revealed increased *Tgfb1* mRNA levels in ALDO systemic GC-A KO mice, and this upregulation was significantly reduced by FR167653 (Supplementary Fig. S2a). Then, we examined the expression of genes associated with inflammation. We examined gene expression relevant to macrophages: *Ccl2* (MCP1) is one of macrophage chemoattractant molecules, and *Adgre1* (F4/80) is one of markers of macrophages. Expression of *Ccl2* (MCP-1) mRNA was increased in ALDO systemic GC-A KO mice, and administration of FR167653 significantly reduced *Ccl2* mRNA levels (Supplementary Fig. S2b). Consistent with this result, FR167653 also reduced *Adgre1* (F4/80) mRNA expression in ALDO systemic GC-A KO mice (Supplementary Fig. S2c). A similar trend in *Cybb* (NOX-2) mRNA expression was observed (Supplementary Fig. S2d).

### Inhibition of p38 MAPK reduces podocyte injury in ALDO systemic GC-A KO mice

**Figure 2:**
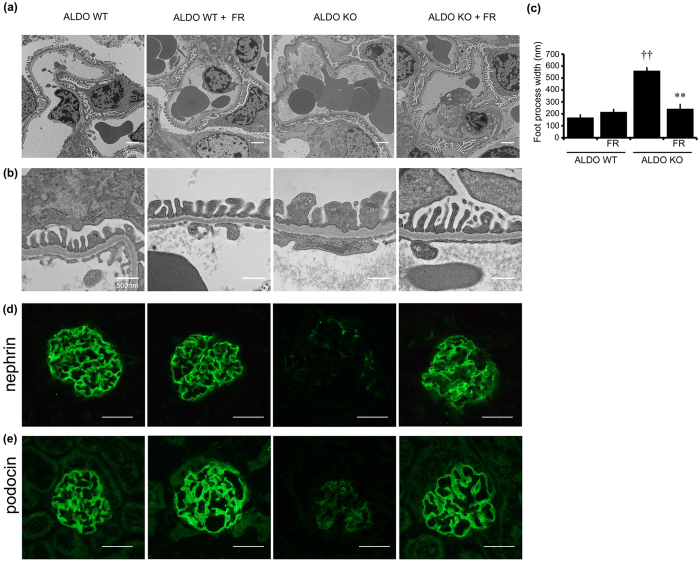
Administration of aldosterone induced podocytes injury, and FR167653 improved podocytes injury. (**a,b**) Electron microscopic photographs of lower (**a**) and higher (**b**) magnification show that treatment with FR167653 in ALDO systemic GC-A KO mice improved GBM thickening and widening foot processes in a superficial glomerulus. Scale bar, 500 nm. (**c**) The width of foot processes in electron microscopic photographs. n = 3. (**d,e**) Representative images of nephrin (**d**) and podocin (**e**) expression by immunofluorescent study in a juxtamedullary glomerulus. Scale bar, 50 μm. ^††^*p* < 0.01, vs. ALDO wild-type mice. **p < 0.01 vs. ALDO systemic GC-A KO mice.

We next evaluated podocyte injury. Electron microscopic analysis revealed that ALDO wild-type mice exhibited slightly widened podocytes foot processes (Fig. 2a,b). ALDO systemic GC-A KO mice demonstrated foot process effacement with irregular thickening of the glomerular basement membrane (GBM), and these changes were improved by FR167653 (Fig. 2a,b). We quantitated the width of foot process and revealed an improvement of foot process effacement by FR167653 (Fig. 2c). Immunofluorescent study demonstrated that reduced nephrin and podocin levels in ALDO systemic GC-A KO mice, and these changes were also ameliorated with administration of a p38 MAPK inhibitor (Fig. 2d,e), indicating that inhibition of the p38 MAPK pathway is closely associated with amelioration of podocyte injury.

**Figure 3:**
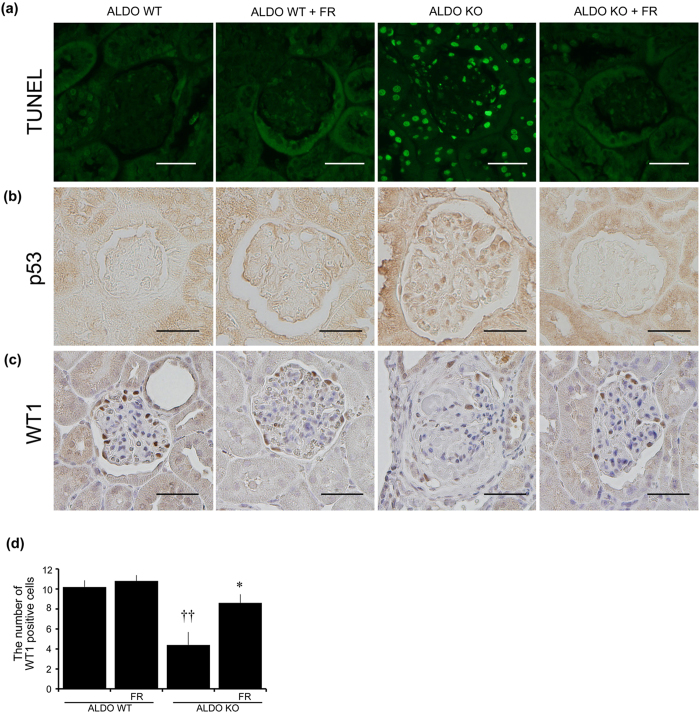
Representative microphotographs of TUNEL, p53, and WT1 staining. (**a**) TUNEL staining shows that TUNEL-positive cells were increased in glomeruli of ALDO systemic GC-A KO mice. Treatment with FR167653 in ALDO systemic GC-A KO mice reduced the number of TUNEL-positive cells. (**b**) Immunohistochemical study for p53. ALDO systemic GC-A KO mice showed increased number of p53-positive cells in glomeruli. Administration with FR167653 reduced the number of p53-positive cells. (**c**) Immunohistochemical study for WT1 in juxtamedullary glomeruli. (**d**) The number of WT1-positive cells in juxtamedullary glomeruli. ALDO systemic GC-A mice showed reduced number of WT1-positive cells, and its decrease was ameliorated with FR167653. Scale bar, 50 μm. Mean ± SEM. ^††^*p* < 0.01, vs. ALDO wild-type mice. *p < 0.05 vs. ALDO systemic GC-A KO mice.

We next examined glomerular apoptosis. ALDO wild-type mice demonstrated almost no apoptotic cells within the glomeruli (Fig. 3a). In contrast, ALDO systemic GC-A KO mice had a large number of TUNEL-positive cells within the glomeruli and tubules, and this increase in the number of apoptotic cells was significantly inhibited by treatment with FR167653 (Fig. 3a). We also examined glomerular p53 expression, as it has been shown to be closely associated with apoptosis^15^. Expression of p53 in glomeruli was not detected in ALDO wild-type mice but was observed in ALDO systemic GC-A KO mice (Fig. 3b). Inhibition of p38 MAPK reduced glomerular expression of p53 in ALDO systemic GC-A KO mice (Fig. 3b). We next counted the number of podocytes by immunohistochemical analysis for Wilms’ tumor 1 (WT1). Administration of FR167653 did not change podocyte numbers in ALDO wild-type mice (Fig. 3c,d). ALDO systemic GC-A KO mice had a significant reduction in the number of podocytes (Fig. 3c,d), and this reduction was reversed by administration of FR167653 (Fig. 3c,d). Phosphorylation of p38 MAPK in glomeruli was augmented in ALDO systemic GC-A KO mice, and this phosphorylation was ameliorated in ALDO systemic GC-A KO mice administered FR167653 (Supplementary Fig. S3). MAPK kinase 3 (MKK3) is an upstream kinase of p38 MAPK^16^. We examined the phosphorylation of MKK3 in these mice by immunohistochemical study. ALDO systemic GC-A KO mice showed enhanced phosphorylation of MKK3 in glomeruli, and this phosphorylation was reduced with the treatment of FR167653 (Supplementary Fig. S4). These results indicate that inhibition of p38 MAPK ameliorates podocyte injury and apoptosis.

### Deletion of GC-A in podocytes aggravates glomerular injury

**Figure 4:**
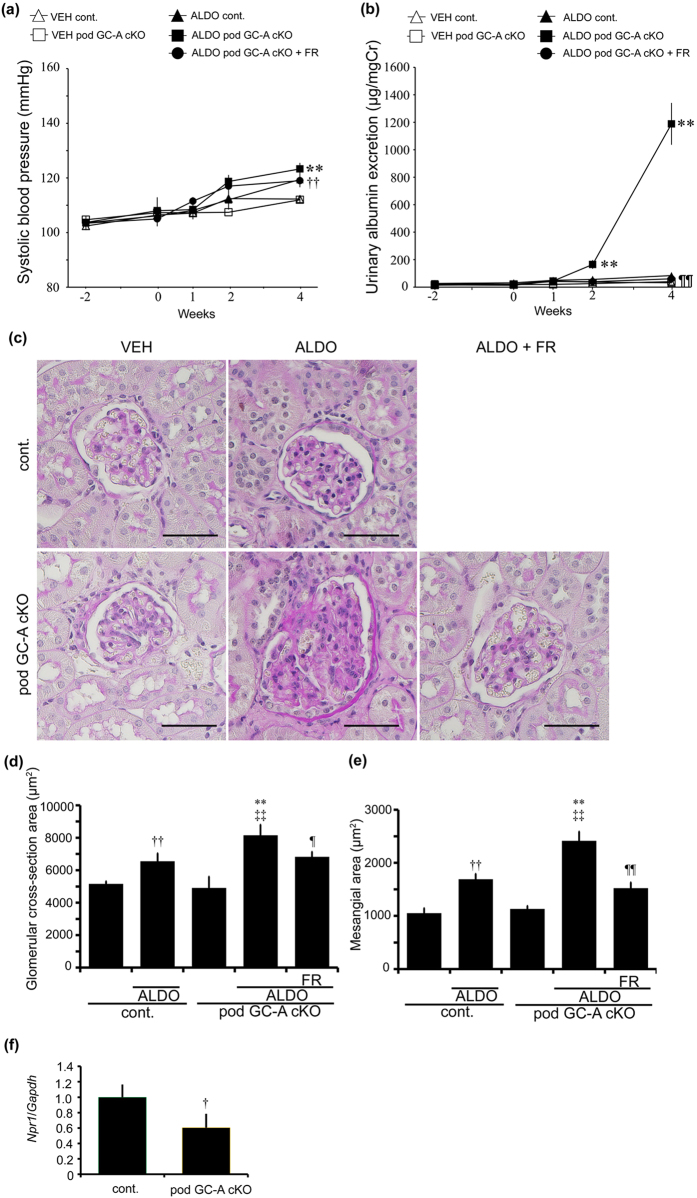
Administration of aldosterone increased urinary albumin excretion in pod GC-A cKO mice, and FR167653 decreased albuminuria without changing blood pressure. (**a**) Time course of systolic blood pressure of VEH control mice, ALDO control mice, VEH pod GC-A cKO mice, ALDO pod GC-A cKO mice, and ALDO pod GC-A cKO mice with FR167653 is shown. Administration of aldosterone increased systemic blood pressure at comparable levels in both control mice and pod GC-A cKO mice. Administration with FR167653 did not change blood pressure. (**b**) Time course of urinary albumin excretion in these five groups. (**c**) PAS staining of renal section were performed at 4 weeks after aldosterone administration. In juxtamedullary glomeruli, ALDO pod GC-A cKO mice showed mesangial expansion with glomerular hypertrophy. Treatment with FR167653 improved these changes. Scale bar, 50 μm. (**d**) Glomerular cross-sectional area and (**e**) mesangial area in juxtamedullary glomeruli at 4 weeks were quantified in five groups. (**f**) Glomerular mRNA expression of *Npr1* in VEH control and pod GC-A cKO mice.n = 7, each. Mean ± SEM. ^†^*p* < 0.05, ^††^*p* < 0.01 vs. VEH control mice, **p < 0.01 vs. VEH pod GC-A cKO mice, ^¶¶^*p* < 0.01 vs. ALDO pod GC-A cKO mice.

Although inhibition of p38 MAPK in ALDO systemic GC-A KO mice ameliorated podocyte and glomerular injury, the relevance of p38 MAPK inhibition with natriuretic peptides in podocyte injury remains obscure. To reveal the role of p38 MAPK signaling in podocytes of ALDO systemic GC-A KO mice, we generated podocyte-specific (pod) GC-A conditional KO (cKO) mice by crossing Nephrin-Cre mice with GC-A floxed mice, and then administering FR167653. Vehicle (VEH)-treated mice were also uninephrectomized, and administered 6% high salt diet and ethanol alone instead of ethanol-containing aldosterone. GC-A floxed mice without Cre recombinase were used as control mice. VEH control and pod GC-A cKO mice had normal SBP (Fig. 4a). ALDO control mice exhibited an increase in SBP of 10 mmHg at four weeks, and ALDO pod GC-A cKO mice had comparable SBP to ALDO control mice (123.2 ± 2.2 vs. 119.4 ± 2.3 mmHg; Fig. 4a). FR167653 was administered to pod GC-A cKO mice as the activation of p38 MAPK is reportedly involved in glomerular injury in ALDO systemic GC-A KO mice. No change in SBP was observed following the administration of FR167653 in ALDO pod GC-A cKO mice (119.0 ± 2.4 mmHg; Fig. 4a). Body weights, kidney weights and serum and urinary parameters are presented in Table 2. Pod GC-A cKO mice exhibited normal kidney weights comparable with control mice (Table 2). ALDO control and pod GC-A cKO mice exhibited renal hypertrophy, and administration of FR167653 had no effect on renal hypertrophy in pod GC-A cKO mice (Table 2). Pod GC-A cKO mice had similar serum and urinary potassium levels to control mice, and aldosterone infusion increased urinary potassium levels and decreased serum potassium levels to the same extent in both control and pod GC-A cKO mice (Table 2). There was no significant difference in serum creatinine and urea nitrogen (UN) levels between ALDO control and pod GC-A cKO mice.

At basal levels, urinary albumin excretion was not different between VEH control mice and pod GC-A cKO mice (18.5 ± 3.3 and 14.2 ± 2.6 μg/mgCr, respectively; Fig. 4b). ALDO control mice had a threefold increase in urinary albumin excretion at four weeks (84.0 ± 6.5 vs. 31.3 ± 3.6 μg/mgCr; Fig. 4b). Of note, ALDO pod GC-A cKO mice demonstrated a significantly increased urinary albumin excretion by 13-fold compared to ALDO control mice at four weeks (84.0 ± 6.5 vs. 1,188 ± 152 μg/mgCr; Fig. 4b). These results indicate that deletion of GC-A in podocytes aggravates aldosterone-induced renal injury. Treatment of ALDO pod GC-A cKO mice with FR167653 suppressed urinary albumin excretion by 95% compared with ALDO pod-GC-A cKO mice (80.5 ± 10.5 μg/mgCr; Fig. 4b), indicating the importance of p38 MAPK pathway in aldosterone-induced podocyte injury.

Pod GC-A cKO mice had normal glomerular histology at baseline. In superficial glomeruli, there was no significant difference in glomerular cross-sectional or mesangial areas between ALDO control and ALDO pod GC-A cKO mice (Supplementary Fig. S5a,b). In juxtamedullary glomeruli, ALDO control mice had mild glomerular hypertrophy and mesangial expansion (Fig. 4c–e). ALDO pod GC-A cKO mice were observed to have marked mesangial expansion and glomerular hypertrophy (Fig. 4c–e). Administration of FR167653 ameliorated glomerular hypertrophy and mesangial expansion (Fig. 4c–e). We examined *Npr1* mRNA to examine the deletion efficacy of *Npr1* gene. Glomerular mRNA expression of *Npr1* (GC-A) in VEH pod GC-A cKO mice was decreased by 40% compared with that in VEH control mice (Fig. 4f).

### Deletion of GC-A in podocytes aggravates podocyte injury and its injury is reversed by inhibition of p38 MAPK

**Figure 5:**
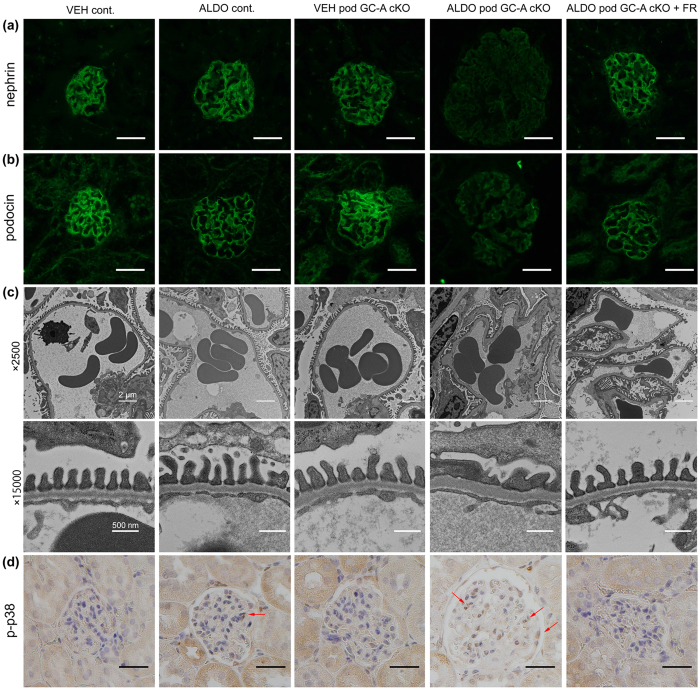
Administration of aldosterone aggravated podocyte injury. (**a,b**) Representative images of nephrin (**a**) and podocin (**b**) expression by immunofluorescent study in a juxtamedullary glomerulus. Scale bars, 50 μm. (**c**) Transmission electron microscopic microphotographs of a juxtamedullary glomerulus. ALDO pod GC-A cKO mice exhibited mild thickening GBM and foot process effacement and its injury was ameliorated with the administration of FR167653. (**d**) Immunohistochemical staining for phosphorylated p38 MAPK. Arrows, phospho-p38 MAPK positive cells. Scale bars, 50 μm.

To evaluate podocyte injury, we performed immunofluorescent analyses of juxtamedullary glomeruli for nephrin and podocin. ALDO control mice exhibited no difference in nephrin and podocin expression compared with VEH control mice (Fig. 5a,b). Although VEH pod GC-A cKO mice had normal nephrin and podocin staining, ALDO pod GC-A cKO mice demonstrated decreased staining intensity for nephrin and podocin (Fig. 5a,b). ALDO pod GC-A cKO mice with FR167653 maintained similar nephrin and podocin staining intensities as VEH pod GC-A cKO mice (Fig. 5a,b). We examined podocyte injury in juxtamedullary glomeruli by electron microscopic analysis. VEH pod GC-A cKO mice demonstrated normal foot processes and GBM (Fig. 5c). ALDO pod GC-A cKO mice exhibited mild thickening of the GBM and foot process effacement, and these changes were improved with the administration of FR167653 (Fig. 5c). Next, we examined glomerular p38 MAPK phosphorylation. No glomerular p38 MAPK phosphorylation was observed in VEH pod GC-A cKO mice (Fig. 5d). Administration of aldosterone much increased phosphorylation of p38 MAPK, particularly in podocytes in pod GC-A cKO mice compared to control mice (Fig. 5d). Phosphorylation of p38 MAPK in glomeruli was reduced by administration of FR167653 (Fig. 5d).

**Figure 6:**
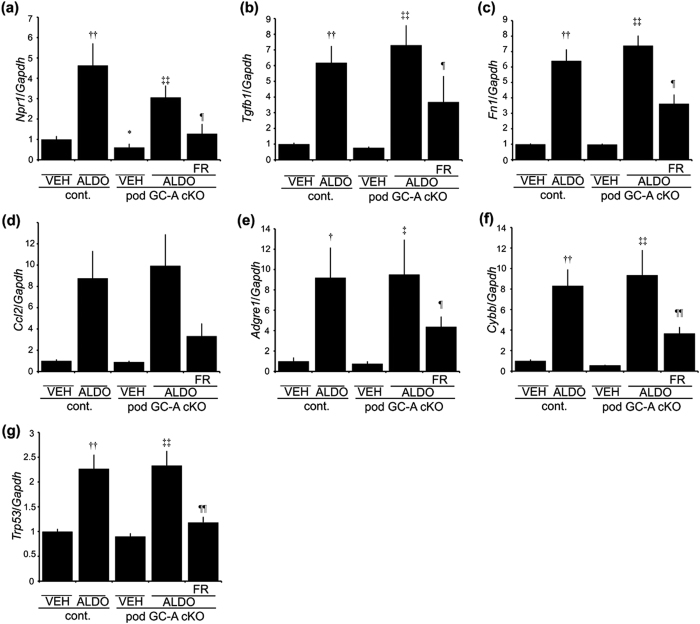
Glomerular mRNA expression at 4 weeks after aldosterone administration. Real-time RT-PCR analysis of *Npr1* (**a**; GC-A), *Tgfb1* (**b**; TGF-β1), *Fn1* (**c**; Fibronectin), *Ccl2* (**d**; MCP-1), *Adgre1* (**e**; F4/80), *Cybb* (**f**; Cybb), *Trp53* (**g**; p53) are shown. Mean ± SEM. ^††^*p* < 0.01, vs. VEH control mice. ^‡^*p* < 0.05, vs. VEH control mice. ^‡‡^*p* < 0.01 vs. VEH pod GC-A cKO mice. ^¶¶^*p* < 0.01 vs. ALDO pod GC-A cKO mice.

We next analyzed glomerular mRNA expression levels. As a previous report showed that natriuretic peptide counteracts aldosterone-induced renal injury^10^, we examined the expression level of *Npr1* in this model. Administration of aldosterone upregulated *Npr1* expression in both control mice and pod GC-A cKO mice, with no significant difference between the two groups (Fig. 6a). FR167653 treatment reduced *Npr1* mRNA expression by 60% (Fig. 6a). We next examined the expression of *Tgfb1* and *Fn1*, because fibronectin (*Fn1*) is one of main extracellular matrix in the kidney and these gene expression is relevant to extracellular matrix accumulation. The expression of extracellular matrix-related gene, such as *Tgfb1* and *Fn1*, was enhanced with administration of aldosterone both in control mice and pod GC-A cKO mice, and was ameliorated by treatment with FR167653 (Fig. 6b,c). We examined gene expression relevant to macrophages: *Ccl2* (MCP1) is one of macrophage chemoattractant molecules, and *Adgre1* (F4/80) is one of markers of macrophages. Expression patterns of *Ccl2* and *Adgre1* were similar to expression levels of *Tgfb1* and *Fn1* (Fig. 6d,e). We next examined the expression of oxidative stress-related genes and downstream target genes of p38 MAPK. Aldosterone also upregulated *Cybb* and *Trp53* (p53) mRNA levels in both control mice and pod GC-A cKO mice, and FR167653 ameliorated these changes (Fig. 6f,g).

**Figure 7:**
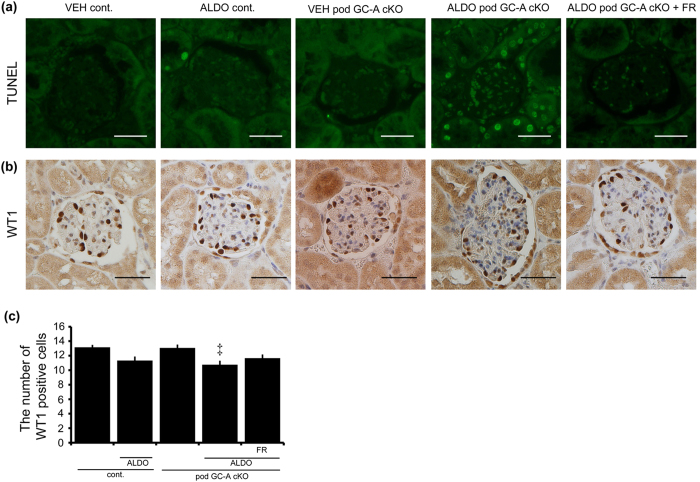
Administration of aldosterone increased apoptosis of podocytes of juxtamedullary glomeruli. (**a**) The number of TUNEL-positive cells was increased in ALDO pod GC-A cKO mice. Administration of FR167653 decreased the number of TUNEL-positive cells in ALDO pod GC-A cKO mice. (**b**,**c**) Administration of aldosterone decreased the number of WT1-positive cells in ALDO pod GC-A cKO mice. Inhibition of p38 MAPK maintained the number of WT1-positive cells. Mean ± SEM. ^‡^*p* < 0.05 vs. VEH pod GC-A cKO mice. Scale bar, 50 μm.

Next, we performed TUNEL assays to evaluate podocyte apoptosis. The number of TUNEL-positive cells in the juxtamedullary glomeruli was increased in ALDO pod GC-A cKO mice (Fig. 7a). Treatment with FR167653 completely abolished TUNEL-positive cells in the glomeruli of ALDO pod GC-A cKO mice (Fig. 7a). The number of WT1-positive cells in superficial glomeruli was not different among groups (Supplementary Fig S6). The number of WT1-positive cells in the juxtamedullary glomeruli of VEH pod GC-A cKO mice did not differ from VEH control mice (Fig. 7b,c). Administration of aldosterone decreased the number of WT1-positive cells in pod GC-A cKO mice compared with pod GC-A cKO mice that were not treated with aldosterone, and this decrease tended to be ameliorated by FR167653 (Fig. 7b,c).

### Natriuretic peptides inhibit phosphorylation of p38 MAPK via activating MKP-1

**Figure 8:**
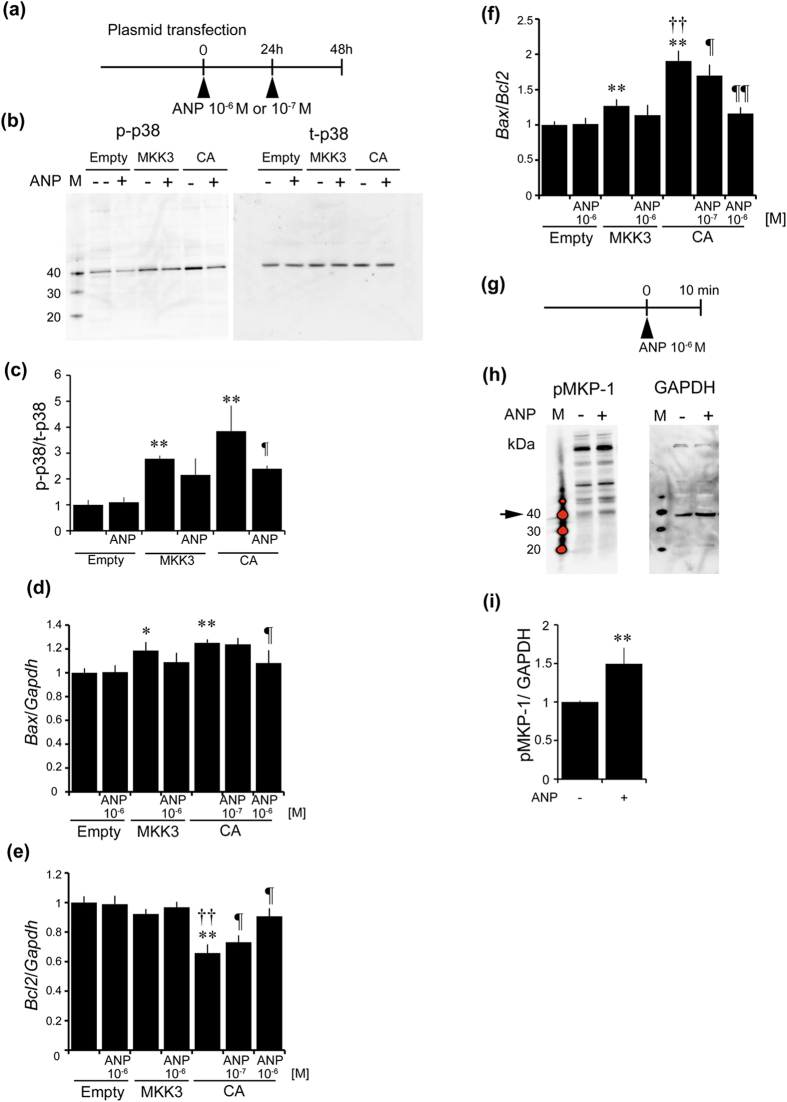
Effect of ANP against podocyte apoptosis induced by activation of p38 MAPK in cultured human podocytes. (**a**) Experimental protocol. We used (1) empty plasmids, (2) plasmids expressing MKK3 (MKK3; pRc/RSV Flag MKK3), or (3) plasmids expressing constitutive active MKK3 (CA; pRc/RSV Flag MKK3 (glu)). MKK3 is an upstream kinase of p38 MAPK. ANP was exchanged at every 24 h. (**b**,**c**) Western blotting of phosphorylation of p38 MAPK and total p38 MAPK in human podocytes by transfection with empty, MKK3 or CA plasmids with or without ANP at 48 h after transfection. (n = 4, each group). Mean ± SEM. **p < 0.01 vs. human podocytes transfected with empty plasmids without ANP, ^¶^*p* < 0.05 vs. CA plasmids without ANP. (**d**–**f**) Real-time RT-PCR analysis for *Bax* (**d**), *Bcl2* (**e**) were examined (n = 6, each group), and *Bax/Bcl2* ratio are shown (**f**). Mean ± SEM. **p < 0.01, *p < 0.05 vs. human podocytes transfected with empty plasmids without ANP, ^††^*p* < 0.01 vs. MKK3 plasmids without ANP, ^¶¶^*p* < 0.01 vs. CA plasmids without ANP. (**g**) Phosphorylation of MKP-1 in human cultured podocytes stimulated with ANP. (**h**,**i**) ANP increased phosphorylation of MKP-1 (arrow) in human podocytes. Glyceraldehyde-3-phosphate dehydrogenase (GAPDH) was used as control. Mean ± SEM. **p < 0.01 vs. human podocytes with vehicle. M, MagicMark XP Western Protein standard, a molecular weight marker.

We next investigated whether ANP can inhibit expression of apoptosis-related proteins induced by activation of p38 MAPK in cultured human podocytes. ANP was administered to the human podocytes transfected with plasmids expressing wild-type MKK3 (MKK3) or constitutive active MKK3 (CA) (Fig. 8a). Phosphorylation of p38 MAPK was increased with following transfection of MKK3 or CA plasmids, and this increase was abolished not by treatment with ANP in MKK3-transfected podocytes, but by treatment with ANP in CA-transfected podocytes (Fig. 8b,c). Expression of *Bax* was not significantly different in MKK3-transfected podocytes, but significantly upregulated in cultured podocytes transfected with CA, and this increase was ameliorated only with high dose ANP (Fig. 8d). Consistent with this result, *Bcl2* mRNA expression was reduced by transfection with CA, and ANP ameliorated this effect in cultured podocytes (Fig. 8e). As a result, the *Bax/Bcl2* ratio was increased following transfection with MKK3 or CA plasmids, and this increase was ameliorated with ANP (Fig. 8f). To confirm the mechanism of ANP-mediated inhibition of p38 MAPK phosphorylation, we examined the phosphorylation status of MAPK phosphatase-1 (MKP-1) which dephosphorylates p38 MAPK, in cultured podocytes (Fig. 8g). Administration of ANP increased phosphorylation of MKP-1 in cultured human podocytes (Fig. 8h,i), indicating that ANP dephosphorylates p38 MAPK via activation of MKP-1.

## Discussion

The present study demonstrated that blockade of p38 MAPK ameliorates glomerular injury in ALDO systemic GC-A KO mice. As we previously reported, ALDO wild-type mice show mild albuminuria, a slight increase in mesangial area, and mild podocyte injury; however, ALDO systemic GC-A KO mice show massive albuminuria with prominent mesangial expansion and podocyte injury^10^. One of the differences between ALDO wild-type and ALDO systemic GC-A KO mice was the phosphorylation status of p38 MAPK, because these two types of mice were found to have similar mRNA expression of inflammation, profibrotic, and oxidative stress genes^10^. The present study clearly demonstrated that the blockade of p38 MAPK reduced albuminuria, mesangial expansion, podocyte injury and apoptosis of glomerular cells in ALDO systemic GC-A mice. Previous studies have reported that blockade of p38 MAPK reduces glomerular injury in several experimental glomerulopathy models, including adriamycin nephropathy^12^, complement-mediated glomerular injury^17^, anti-GBM glomerulonephritis^13^, lupus nephritis^18^, thrombotic microangiopathy^19^, and diabetic nephropathy^20^. However, the role of p38 MAPK activation in response to aldosterone has yet to be fully elucidated, especially in the glomeruli. The present study has unveiled the novel pathway of aldosterone-induced glomerular injury. Aldosterone has been shown to promote apoptosis in many cell types, including podocytes through phosphoinositide-3/Akt, p38 MAPK and p53 pathways *in vitro*^21^. However, the role of p38 MAPK in aldosterone-induced podocyte apoptosis remains elusive. The present study demonstrates that blockade of p38 MAPK ameliorates apoptosis in podocytes, potentially indicating the mechanism by which MAPK signaling reduces podocyte injury.

Interestingly, administration of FR167653 reduced systolic blood pressure in ALDO systemic GC-A KO mice by 20 mmHg, although it had no effect on systolic blood pressure in ALDO wild-type mice. ALDO systemic GC-A KO mice had similar levels of serum aldosterone, and daily urinary sodium excretion, suggesting that the reduction in systolic blood pressure was not due to increased sodium excretion. Other possible explanations are changes in blood vessel tone and cardiac output. Tojo *et al*. reported that administration of FR167653 to Dahl salt-sensitive rats reduces expression of interleukin-1β and tumor necrosis factor-α in the whole kidney without altering blood pressure^22^. However, Potthoff *et al*. recently reported that chronic p38 MAPK inhibition improves vascular function and reduces systolic blood pressure in angiotensin II-stimulated hypertension in mice^23^. Interestingly, they also reported that non-treated mice do not exhibit blood pressure reduction^23^, consistent with our results.

Kuhn *et al*. reported that BNP activates phosphorylation of p38 MAPK in microvascular endothelial cells^24^, a finding contradictory to our results. However, phosphorylation of p38 MAPK is context-dependent rather than ligand-dependent. Phosphorylation of p38 MAPK is prominent in podocytes in ALDO systemic GC-A KO mice; therefore, decreased p38 MAPK activity in response to FR167653 appears to function in the protection of podocytes. However, we were unable to exclude the effects of blood pressure reduction and the role of mesangial cells as well as endothelial cells in ALDO systemic GC-A KO mice administered FR167653. To overcome these limitations, we generated podocyte-specific GC-A cKO mice and administered aldosterone and high salt following uninephrectomy. Recently, Staffel *et al*. reported that uninephrectomized pod GC-A cKO mice with deoxycorticosterone acetate and high salt diet exhibit similar blood pressure and increased albuminuria compared with control mice, confirming the protective role of natriuretic peptides/GC-A pathway in podocytes^25^. We also constructed pod GC-A cKO mice and demonstrated increased urinary albumin and glomerular injury without blood pressure elevation in ALDO pod GC-A cKO mice. Especially, juxtamedullary glomeruli rather than superficial glomeruli exhibited prominent mesangial expansion and podocyte injury in ALDO pod GC-A cKO mice. We speculate that hemodynamic changes will be the important factor on glomerular injury in ALDO pod GC-A cKO mice, because aldosterone concentration should be same between superficial and juxtamedullary glomeruli. Administration of FR167653 to ALDO pod GC-A cKO mice ameliorated albuminuria, mesangial expansion, loss of nephrin and podocin, and podocyte foot process effacement, suggesting that podocytes are one of the main targets of FR167653. Several studies showed genetic deletion of a specific molecule in podocytes affects mesangial expansion^26^,^27^, probably because of paracrine effects between podocytes and mesangial cells. However, GC-A was localized in mesangial cells^28^ and endothelial cells^29^ as well as in podocytes. In addition, urinary albumin excretion of ALDO pod GC-A cKO mice was much less than that of ALDO systemic GC-A KO mice, although ALDO pod GC-A cKO mice showed lower SBP. These finding may indicate the importance of mesangial or endothelial cells in the signaling of natriuretic peptides. This is the limitation of this study.

Aldosterone has been shown to phosphorylate p38 MAPK in many cell types, including vascular smooth muscle cells^30^, cardiomyocytes^31^, neutrophils^32^, peritoneal mesothelial cells^33^, and podocytes^21^. However, the relationship between natriuretic peptides and phosphorylation of p38 MAPK is cell-type and context-dependent. Previous studies have reported that natriuretic peptides activates p38 MAPK in hypoxia-exposed hepatocytes^34^, lung endothelial cells^24^ and brown adipocytes^35^. In contrast, other reports, including our previous study, have demonstrated that natriuretic peptides abolish p38 MAPK activation induced by inflammatory stimuli in human umbilical endothelial cells^36^, bovine pulmonary microvascular endothelial cells^37^, fibroblasts^38^ and podocytes^10^. The mechanism by which natriuretic peptides reduce phosphorylation of p38 MAPK has yet to be fully evaluated; however, a study has reported that natriuretic peptides augment MKP-1, which dephosphorylates p38 MAPK, in endothelial cells^39^. Consistent with this study, the present study also demonstrated the MKP-1 phosphorylation following treatment of podocytes with ANP. These results indicate that deletion of GC-A enhances phosphorylation of p38 MAPK in the kidney following stimulation with aldosterone.

Previous studies have reported that aldosterone induces apoptosis in mesangial cells^40^, endothelial cells^41^, renal tubular cells^42^, and podocytes^43^ in the kidney. Natriuretic peptides have been documented to have activity against apoptosis in cells in renal cortex and medulla in SHR rats^44^, macrophages^45^, cardiomyocytes in *db/db* mice^46^, and hepatocytes^47^. Conversely, other reports have shown that ANP induces apoptosis^48^,^49^. However, the contribution of natriuretic peptides to apoptosis in podocytes remains unclear. In the present study, we demonstrate that natriuretic peptides inhibit *Bax* expression, leading to decreased levels of apoptosis.

In conclusion, inhibition of p38 MAPK pathway ameliorates glomerular injury in uninephrectomized, high-salt fed, and aldosterone-infused mice by protecting against podocyte injury. These results indicate that p38 MAPK inhibitors may represent promising therapeutic targets against renal injury.

## Methods

### Reagents and antibodies

Aldosterone was obtained from Sigma Aldrich (St. Louis, MO). FR167653, p38α MAP Kinase inhibitor, was kindly provided by Astellas Pharma Inc. (Tokyo, Japan). Primary antibodies used for immunohistochemical studies and Western blotting were goat anti-nephrin (R&D Systems, Minneapolis, MN), rabbit anti-podocin (Sigma Aldrich), rabbit anti-phospho-p38 MAPK (Cell Signaling Technology, Boston, MA), rabbit total p38 MAPK (Cell Signaling Technology), rabbit anti-WT1 (Santa Cruz, Dallas, TX), and rabbit anti-p53 (Vector Laboratories, Burlingame, CA), rabbit anti-phospho-MKP-1 (Cell Signaling Technology), rabbit anti-phospho-MKK3 (Cell Signaling Technology), and mouse anti-glyceraldehyde-3-phosphate dehydrogenase (GAPDH; Santa Cruz) antibodies. pRc/RSV Flag MKK3^50^ and pRc/RSV Flag MKK3 (glu)^51^ were gifts from Professor Roger Davis (Addgene plasmid #14671 and #14670, respectively).

### Animal experiments

All animal experiments were performed in accordance with Fundamental Guidelines for Proper Conduct of Animal Experiment and Related Activities in Academic Research Institutions and were approved by the Animal Experimentation Committee of Kyoto University Graduate School of Medicine. Male systemic GC-A KO mice^52^ or their wild-type littermates received left uninephrectomy under intraperitoneal pentobarbital anesthesia and osmotic minipump (ALZET 2004, Cupertino, CA) implantation subcutaneously to infuse aldosterone (0.2 μg/kg body weight per minute) or vehicle. All mice were fed a diet containing 6% NaCl^10^. FR167653 was administered by dissolving it in drinking water^12^. Blood pressure was measured indirectly by using the tail-cuff method (MK-2000ST; Muromachi Kikai, Tokyo, Japan) at −2, 0, 1, 2, and 4 weeks. Urine samples were collected from metabolic cages (Shinano manufacturing, Tokyo, Japan) for 24 h at −2, 0, 1, 2, and 4 weeks and urine volumes were measured. Blood and kidney samples were harvested at 4 weeks. Urinary albumin levels were measured with albumin ELISA kits (Exocell, Philadelphia, PA). Serum and urinary Cr, sodium, and potassium levels were measured by enzymatic methods (SRL, Tokyo, Japan).

Construction of GC-A floxed mice and nephrin-promotor driven Cre recombinase transgenic mice were as described previously^53^,^54^. By crossing GC-A floxed mice (C57BL/6 J background) with Nephrin Cre mice (C57BL/6 J background), we generated pod GC-A cKO mice (*Npr1*^fl/fl^, Nephrin Cre/+mice) and control littermates (*Npr1*^fl/fl^,+/+). Male pod GC-A cKO mice or control mice at 12 weeks of age were uninephrectomized, and administered with 6% NaCl diet and aldosterone or vehicle using the same protocol for systemic GC-A KO mice.

### Renal histology and electron microscopy

Histological and electron microscopic examinations were performed as described previously^55^. Periodic acid-Schiff (PAS) stained kidney samples were examined by light microscopy. Mesangial areas in juxtamedullary glomeruli were measured using MetaMorph Software (Molecular Devices, Sunnyvale, CA). Electron microscopic examination was performed using an electron microscope (H-7600, Hitachi, Tokyo, Japan).

### Immunohistochemistry

Immunofluorescent studies for nephrin and podocin were performed as descried previously^10^. Briefly, cryostat sections were incubated with goat anti-nephrin antibody or rabbit anti-podocin antibody, and then incubated with fluorescein isothiocyanate (FITC)-labeled secondary antibody. Immunohistochemical analysis for phospho-p38 MAPK, WT1, p53, phospho-MKK3 were as previously described with some modifications^10^. Staining for the presence of apoptotic cells was performed using *in situ* cell death detection kits (Roche, Mannheim, Germany). Paraffin-embedded renal sections were reacted with fluorescent dyed mixed solutions.

### Cell culture and transfection

Conditionally immortalized human podocytes were provided by Dr. Moin Saleem, and cultured and differentiated as described previously^56^,^57^. Briefly, cells were cultured with RPMI 1640 medium (Sigma-Aldrich) supplemented with 10% fetal bovine serum (FBS; Biowest, Nuaille, France) and insulin-transferrin-selenite supplement (LifeTechnologies, Carlsbad CA). Differentiated podocytes were transfected with pRc/RSV Flag MKK3, pRc/RSV Flag MKK3 (glu) or empty plasmid using Nucleofector Kit for primary Mammalian Epithelia cells (Lonza, Basel, Switzerland) as described previously^57^. Transfected cells were incubated with vehicle or ANP, with the exchange of the medium containing ANP at every 24 h, and then harvested for RNA and protein analysis at 48 h. To confirm phosphorylation of MKP-1, differentiated podocytes were treated with ANP for 10 min and then harvested.

### Glomerular RNA, protein extraction and real-time RT-PCR

Glomeruli were isolated by the graded sieving methods as described previously^10^. RNA and protein extraction was performed using AllPrep DNA/RNA/protein kits (QIAGEN, Hilden, Germany). Quantitative real-time PCR was performed using StepOnePlus System (Thermo Fischer Scientific, Waltham, MA), as descried previously^58^. *Npr1, Tgfb1, Ccl2, Fn1, Cybb, Adgre1, Trp53, Bax*, and *Bcl2* mRNA expression levels were evaluated. Some primer and probe sets were described previously^10^. *Adgre1* forward primer, 5′-tggtggtcataatctctgcttctg-3′; *Adgre1* reverse primer, 5′-agacaggccccaggaaactc-3′; *Adgre1* probe, 5′-FAM-cccgtctctgtattcaaccagcagcgatt-TAMRA-3′; *Trp53* forward primer, 5′-gcttctccgaagactggatgac-3′; *Trp53* reverse primer, 5′-gcagtgaggtgatggcagg-3′; *Trp53* probe primer, 5′-FAM-tcacagtcggatatcagcctcgagctcc-TAMRA-3′; *Bax* forward primer, 5′-aactggtgctcaaggccct-3′; *Bax* reverse primer, 5′-cccggaggaagtccaatgtc-3′; *Bax* probe primer, 5′-tgcaccaaggtgccggaactgatca-TAMRA-3′; *Bcl2* forward primer, 5′-gatgggatcgttgccttatgca-3′; *Bcl2* reverse primer, 5′-cagtctacttcctctgtgatgttgta-3′; 5′-cagcatgatcctctgtcaagtttcctt-TAMRA-3′, *Bcl2* probe primer, 5′-FAM-cagcatgatcctctgtcaagtttcctt -TAMRA-3′.

### Western blotting

Western blotting was performed as previously described^10^. Filters on isolated cell extracts were incubated with rabbit anti-phospho p38 MAPK antibody, rabbit anti-p38 MAPK antibody, rabbit anti-phospho MKP-1 antibody, or mouse anti-GAPDH antibody. Immunoblot were developed using horseradish peroxidase-linked donkey anti-rabbit or anti-mouse antibody and a chemiluminescent kit.

### Statistical Analysis

Data are expressed as the mean ± SE. Statistical analysis was performed using ANOVA or Student’s t test as appropriate.

## Additional Information

**How to cite this article**: Kato, Y. *et al*. Natriuretic peptide receptor guanylyl cyclase-A pathway counteracts glomerular injury evoked by aldosterone through p38 mitogen-activated protein kinase inhibition. *Sci. Rep.* **7**, 46624; doi: 10.1038/srep46624 (2017).

**Publisher's note:** Springer Nature remains neutral with regard to jurisdictional claims in published maps and institutional affiliations.

## Supplementary Material

Supplementary Figures

## Figures and Tables

**Table 1:** Body and kidney weights, and blood and urinary parameters in ALDO wild-type or ALDO systemic GC-A KO mice with or without FR167653.

	ALDO WT	ALDO WT + FR	ALDO KO	ALDO KO + FR
BW at 4 wk, g	29.1 ± 0.4	29.1 ± 0.5	28.9 ± 0.8	28.0 ± 0.5
LKW at 4 wk, mg	312 ± 13	322 ± 16	396 ± 7^††^	364 ± 16
LKW/BW at 4 wk, mg/g	10.75 ± 0.64	11.07 ± 0.62	13.70 ± 0.44^††^	13.04 ± 0.84
Serum UN, mg/dL	23.9 ± 1.9	23.9 ± 1.3	23.5 ± 3.4	24.0 ± 1.9
Serum Cr, mg/dL	0.142 ± 0.012	0.166 ± 0.017	0.166 ± 0.029	0.170 ± 0.008
Serum sodium, mEq/L	162.2 ± 1.0	160.4 ± 0.9	162.6 ± 1.3	160.2 ± 1.5
Serum potassium, mEq/L	3.02 ± 0.21	3.46 ± 0.12	3.10 ± 0.10	3.36 ± 0.22
Urinary sodium, mEq/day	2.26 ± 0.39	1.90 ± 0.09	2.87 ± 0.41	2.50 ± 0.28
Urinary potassium, mEq/day	0.49 ± 0.06	0.54 ± 0.08	0.69 ± 0.07	0.62 ± 0.04
Urinary potassium/sodium	0.246 ± 0.023	0.273 ± 0.020	0.237 ± 0.029	0.251 ± 0.019
Urinary albumin, μg/mgCr	73.9 ± 9.5	39.0 ± 4.0	9,268 ± 737^††^	580 ± 99^**^

BW, body weights; LKW, left kidney weights; UN, urea nitrogen; Cr, creatinine.

Values are expressed as the mean ± SEM for aldosterone-infused and high salt diet-fed uninephrectomized (ALDO) wild-type mice (ALDO WT; n = 5), ALDO wild-type mice with FR167653 (ALDO WT + FR; n = 5), ALDO systemic GC-A KO mice (ALDO KO: n = 5), ALDO systemic GC-A KO mice with FR167653 (ALDO KO + FR; n = 5).

^††^*p* < 0.01, ALDO WT vs. ALDO KO.

**p < 0.01, ALDO KO vs. ALDO KO + FR.

**Table 2:** Body and kidney weights, and blood and urinary parameters in VEH or ALDO control or pod-GC-A cKO mice.

	VEH cont.	ALDO cont.	VEH cKO	ALDO cKO	ALDO cKO + FR
BW at 4 wk, g	29.9 ± 0.3	30.1 ± 0.5	29.2 ± 0.3	30.2 ± 0.4	30.0 ± 0.5
LKW at 4 wk, mg	245 ± 6	304 ± 12^**††**^	241 ± 6	322 ± 9^**^	337 ± 10
LKW/BW at 4 wk, mg/g	8.23 ± 0.18	10.14 ± 0.54^**††**^	8.24 ± 0.16^¶¶^	10.67 ± 0.35^**^	11.24 ± 0.38
Serum UN, mg/dL	37.8 ± 1.9	36.3 ± 2.9	41.35 ± 1.4	30.65 ± 2.2^*^	24.5 ± 1.5
Serum Cr, mg/dL	0.168 ± 0.006	0.196 ± 0.019	0.181 ± 0.008	0.147 ± 0.012	0.147 ± 0.009
Serum sodium, mEq/L	154.1 ± 1.3	160.0 ± 0.9	156.4 ± 1.2	158.1 ± 0.8	159.8 ± 0.6
Serum potassium, mEq/L	4.43 ± 0.12	3.76 ± 0.24	4.67 ± 0.09	3.54 ± 0.23^*^	3.57 ± 0.10
Urinary sodium, mEq/day	3.00 ± 0.18	3.28 ± 0.31	3.10 ± 0.22	3.58 ± 0.31	3.40 ± 0.23
Urinary potassium, mEq/day	0.62 ± 0.03	0.71 ± 0.07^**††**^	0.58 ± 0.04	0.83 ± 0.08^**^	0.71 ± 0.03
Urinary potassium/sodium	0.211 ± 0.005	0.222 ± 0.015	0.188 ± 0.004	0.246 ± 0.032	0.212 ± 0.010
Urinary albumin, μg/mgCr	31.3 ± 3.6	84.0 ± 6.5^†, ¶¶^	40.2 ± 9.7	1,188 ± 152^**^	80.5 ± 10.5^¶^

BW, body weights; LKW, left kidney weights; UN, urea nitrogen; Cr, creatinine.

Values are expressed as the mean ± SEM for vehicle-infused high salt fed uninephrectomized (VEH) control mice (VEH cont.; n = 7), ALDO control mice (ALDO cont.; n = 7), VEH pod GC-A cKO mice (VEH cKO: n = 7), ALDO pod GC-A cKO mice (ALDO cKO; n = 7), ALDO pod GC-A cKO mice with FR167653 (ALDO cKO + FR; n = 7).

^†^*p* < 0.05, ^††^*p* < 0.01, VEH cont. vs. ALDO cont.

*p < 0.05, **p < 0.01, VEH pod GC-A cKO vs. ALDO pod GC-A cKO.

^¶^*p* < 0.05, ^¶¶^*p* < 0.01, vs. ALDO pod GC-A cKO.

## References

[b1] Brown J. N. Contribution of aldosterone to cardiovascular and renal inflammation and fibrosis. Nat. Rev. Nephrol. 9, 459–469 (2013). 23774812 10.1038/nrneph.2013.110 PMC3922409

[b2] Shibata S. . Podocyte as the target for aldosterone: roles of oxidative stress and Sgk1. Hypertension 49, 355–364 (2007). 17200434 10.1161/01.HYP.0000255636.11931.a2

[b3] Harada E. . Aldosterone induces angiotensin-converting-enzyme gene expression in cultured neonatal rat cardiocytes. Circulation 104, 137–139 (2001). 11447075 10.1161/01.cir.104.2.137

[b4] Nishiyama A. . Possible contributions of reactive oxygen species and mitogen-activated protein kinase to renal injury in aldosterone/salt-induced hypertensive rats. Hypertension 43, 841–848 (2004). 14769808 10.1161/01.HYP.0000118519.66430.22

[b5] Nagase M. & Fujita T. Aldosterone and glomerular podocyte injury. Clin. Exp. Nephrol. 12, 233–242 (2008). 18317876 10.1007/s10157-008-0034-9

[b6] Terada Y. . Aldosterone stimulates proliferation of mesangial cells by activating mitogen-activated protein kinase 1/2, cyclin D1, and cyclin A. J. Am. Soc. Nephrol. 16, 2296–2305 (2005). 15975997 10.1681/ASN.2005020129

[b7] Oberleithner H. Aldosterone makes human endothelium stiff and vulnerable. Kidney Int. 67, 1680–1682 (2005). 15840012 10.1111/j.1523-1755.2005.00263.x

[b8] Remuzzi G., Cattaneo D. & Perico N. The aggravating mechanisms of aldosterone on kidney fibrosis. J. Am. Soc. Nephrol. 19, 1459–1462 (2008). 18550649 10.1681/ASN.2007101079

[b9] Nakao K., Ogawa Y., Suga S. & Imura H. Molecular biology and biochemistry of the natriuretic peptide system. I: Natriuretic peptides. J. Hypertens. 10, 907–912 (1992). 1328371

[b10] Ogawa Y. . Natriuretic peptide receptor guanylyl cyclase-A protects podocytes from aldosterone-induced glomerular injury. J. Am. Soc. Nephrol. 23, 1198–1209 (2012). 22652704 10.1681/ASN.2011100985 PMC3380647

[b11] Kumar S., Boehm J. & Lee C. J. p38 MAP kinases: key signalling molecules as therapeutic targets for inflammatory diseases. Nat. Rev. Drug. Discov. 2, 717–726 (2003). 12951578 10.1038/nrd1177

[b12] Koshikawa M. . Role of p38 mitogen-activated protein kinase activation in podocyte injury and proteinuria in experimental nephrotic syndrome. J. Am. Soc. Nephrol. 16, 2690–2701 (2005). 15987752 10.1681/ASN.2004121084

[b13] Stambe C. . Blockade of p38α MAPK ameliorates acute inflammatory renal injury in rat anti-GBM glomerulonephritis. J. Am. Soc. Nephrol. 14, 338–351 (2003). 12538734 10.1097/01.asn.0000048715.12315.fd

[b14] Sheryanna A. . Inhibition of p38 mitogen-activated protein kinase is effective in the treatment of experimental crescentic glomerulonephritis and suppresses monocyte chemoattractant protein-1 but not IL-1β or IL-6. J. Am. Soc. Nephrol. 18, 1167–1179 (2007). 17314328 10.1681/ASN.2006010050

[b15] Pippin W. J. . DNA damage is a novel response to sublytic complement C5b-9-induced injury in podocytes. J. Clin. Invest. 111, 877–885 (2003). 12639994 10.1172/JCI15645 PMC153762

[b16] Derijard B. . Independent human MAP-kinase signal transduction pathways defined by MEK and MKK isoforms. Science 267, 682–685 (1995). 7839144 10.1126/science.7839144

[b17] Aoudjit L., Stanciu M., Li H., Lemay S. & Takano T. p38 mitogen-activated protein kinase protects glomerular epithelial cells from complement-mediated cell injury. Am. J. Physiol. Renal Physiol. 285, F765–774 (2003). 12837681 10.1152/ajprenal.00100.2003

[b18] Iwata Y. . p38 mitogen-activated protein kinase contributes to autoimmune renal injury in MRL-Fas^lpr^ mice. J. Am. Soc. Nephrol. 14, 57–67 (2003). 12506138 10.1097/01.asn.0000037402.83851.5f

[b19] Fu J. X. . Role of p38 MAP kinase pathway in a toxin-induced model of hemolytic uremic syndrome. Pediatr. Nephrol. 19, 844–852 (2004). 15206036 10.1007/s00467-004-1502-4

[b20] Peng L. . The protective effect of beraprost sodium on diabetic nephropathy by inhibiting inflammation and p38 MAPK signaling pathway in high-fat diet/streptozotocin-induced diabetic rats. Int. J. Endocrinol. 2016, 1690474 (2016). 27212945 10.1155/2016/1690474 PMC4860249

[b21] Chen C. . Aldosterone induces apoptosis in rat podocytes: role of PI3-K/Akt and p38MAPK signaling pathways. Nephron Exp. Nephrol. 113, e26–34 (2009). 19590239 10.1159/000228080 PMC2790761

[b22] Tojo A. . Antioxidative effect of p38 mitogen-activated protein kinase inhibitor in the kidney of hypertensive rat. J. Hypertens. 23, 165–174 (2005). 15643139 10.1097/00004872-200501000-00027

[b23] Potthoff A. S. . Chronic p38 mitogen-activated protein kinase inhibition improves vascular function and remodeling in angiotensin II-dependent hypertension. J. Renin Angiotensin. Aldosterone Syst. 17, 1470320316653284 (2016). 27407119 10.1177/1470320316653284 PMC5843849

[b24] Kuhn M. . The natriuretic peptide/guanylyl cyclase-A system functions as a stress-responsive regulator of angiogenesis in mice. J. Clin. Invest. 119, 2019–2030 (2009). 19487812 10.1172/JCI37430 PMC2701863

[b25] Staffel J. . Natriuretic peptide receptor guanylyl cyclase-A in podocytes is renoprotective but dispensable for physiologic renal function. J. Am. Soc. Nephrol. 28, 260–277 (2017). 27153922 10.1681/ASN.2015070731 PMC5198264

[b26] Lenoir O. . Direct action of endothelin-1 on podocytes promotes diabetic glomerulosclerosis. J. Am. Soc. Nephrol. 25, 1050–1062 (2014). 24722437 10.1681/ASN.2013020195 PMC4005294

[b27] Mariya T. . Notch1 and Notch2 in podocytes play differential roles during diabetic nephropathy development. Diabetes 64, 4099–4111 (2015). 26293507 10.2337/db15-0260 PMC4657584

[b28] Tripathi S. & Pandey K. N. Guanylyl cyclase/natriuretic peptide receptor-A signaling antagonizes the vascular endothelial grwoth factor-stimulated MAPKs and downstream effectors AP-1 and CREB in mouse mesangial cells. Mol. Cell. Biochem. 368, 47–59 (2012). 22610792 10.1007/s11010-012-1341-8 PMC3488346

[b29] Chen W. . Atrial natriuretic peptide enhances microvascular albumin permeability by the caveolae-mediated transcellular pathway. Cardiovasc Res 93, 141–151 (2012). 22025581 10.1093/cvr/cvr279 PMC3243041

[b30] Callera E. G. . Aldosterone activates vascular p38MAP kinase and NADPH oxidase via c-Src. Hypertension 45, 773–779 (2005). 15699470 10.1161/01.HYP.0000154365.30593.d3

[b31] Lopez-Andres N., Inigo C., Gallego I., Diez J. & Fortuno A. M. Aldosterone induces cardiotrophin-1 expression in HL-1 adult cardiomyocytes. Endocrinology 149, 4970–4978 (2008). 18566129 10.1210/en.2008-0120

[b32] Walczak C. . Aldosterone increases VEGF-A production in human neutrophils through PI3K, ERK1/2 and p38 pathways. Biochim. Biophys. Acta. 1813, 2125–2132 (2011). 21803079 10.1016/j.bbamcr.2011.07.010

[b33] Yu M. . Effect of aldosterone on epithelial-to-mesenchymal transition of human peritoneal mesothelial cells. Kidney Res. Clin. Pract. 34, 83–92 (2015). 26484027 10.1016/j.krcp.2015.03.005 PMC4570652

[b34] Carini R. . Mechanisms of hepatocyte protection against hypoxic injury by atrial natriuretic peptide. Hepatology 37, 277–285 (2003). 12540777 10.1053/jhep.2003.50033

[b35] Bordicchia M. . Cardiac natriuretic peptides act via p38 MAPK to induce the brown fat thermogenic program in mouse and human adipocytes. J. Clin. Invest. 122, 1022–1036 (2012). 22307324 10.1172/JCI59701 PMC3287224

[b36] Kiemer K. A. . Inhibition of p38 MAPK activation via induction of MKP-1: atrial natriuretic peptide reduces TNF-α-induced actin polymerization and endothelial permeability. Circ. Res., 90, 874–881 (2002). 11988488 10.1161/01.res.0000017068.58856.f3

[b37] Irwin C. D., Patot Tv, C. M, Tucker A. & Bowen R. Direct ANP inhibition of hypoxia-induced inflammatory pathways in pulmonary microvascular and macrovascular endothelial monolayers. Am. J. Physiol. Lung Cell Mol. Physiol. 288, L849–L859 (2005). 15618455 10.1152/ajplung.00294.2004

[b38] Song Z. . Recombinant human brain natriuretic peptide attenuates LPS-induced cellular injury in human fetal lung fibroblasts via inhibiting MAPK and NF-κB pathway activation. Mol. Med. Rep. 14, 1785–1790 (2016). 27314600 10.3892/mmr.2016.5400

[b39] Furst R. . Atrial natriuretic peptide induces mitogen-activated protein kinase phosphatase-1 in human endothelial cells via Rac1 and NAD(P)H oxidase/Nox2-activation. Circ. Res. 96, 43–53 (2005). 15569826 10.1161/01.RES.0000151983.01148.06

[b40] Shi H., Zhang A., He Y., Yang M. & Gan W. Effects of p53 on aldosterone-induced mesangial cell apoptosis *in vivo* and *in vitro*. Mol. Med. Rep. 13, 5102–5108 (2016). 27109859 10.3892/mmr.2016.5156 PMC4878551

[b41] Qiao W., Zhang W., Shao S., Gai Y. & Zhang M. Effect and mechanism of poly (ADP-ribose) polymerase-1 in aldosterone-induced apoptosis. Mol. Med. Rep. 12, 1631–1638 (2015). 25872931 10.3892/mmr.2015.3596 PMC4464439

[b42] Kitada K. . Aldosterone induces p21-regulated apoptosis via increased synthesis and secretion of tumour necrosis factor-α in human proximal tubular cells. Clin. Exp. Pharmacol. Physiol. 39, 858–863 (2012). 23013131 10.1111/1440-1681.12001 PMC3478382

[b43] Yuan Y. . The roles of oxidative stress, endoplasmic reticulum stress, and autophagy in aldosterone/mineralocorticoid receptor-induced podocyte injury. Lab. Invest. 95, 1374–1386 (2015). 26414307 10.1038/labinvest.2015.118

[b44] Romero M. . Chronic treatment with atrial natriuretic peptide in spontaneously hypertensive rats: beneficial renal effects and sex differences. PLoS One 10, e0120362 (2015). 25774801 10.1371/journal.pone.0120362 PMC4361555

[b45] Chang C. . The inhibition of oxidised low-density lipoprotein-induced apoptosis of macrophages by recombinant human brain natriuretic peptide and the underlying mechanism. Cardiology 132, 137–146 (2015). 26278917 10.1159/000433464

[b46] Plante E. . Treatment with brain natriuretic peptide prevents the development of cardiac dysfunction in obese diabetic *db/db* mice. Diabetologia 57, 1257–1267 (2014). 24595856 10.1007/s00125-014-3201-4

[b47] Yamada T., Kotake Y., Nagata H. & Takeda J. Atrial natriuretic peptide reduces hepatic ischemia-reperfusion injury in rabbits. J. Anesth. 27, 901–908 (2013). 23736823 10.1007/s00540-013-1643-3

[b48] Wu F. C., Bishopric H. N. & Pratt E. R. Atrial natriuretic peptide induces apoptosis in neonatal rat cardiac myocytes. J. Biol. Chem. 272, 14860–14866 (1997). 9169455 10.1074/jbc.272.23.14860

[b49] Suenobu N., Shichiri M., Iwashina M., Marumo F. & Hirata Y. Natriuretic peptides and nitric oxide induce endothelial apoptosis via a cGMP-dependent mechanism. Arterioscler. Thromb. Vasc. Biol. 19, 140–146 (1999). 9888876 10.1161/01.atv.19.1.140

[b50] Derijard B. . Independent human MAP-kinase signal transduction pathways defined by MEK and MKK isoforms. Science 267, 682–685 (1995). 7839144 10.1126/science.7839144

[b51] Raingeaud J. . MKK3- and MKK6-regulated gene expression is mediated by the p38 mitogen-activated protein kinase signal transduction pathway. Mol. Cell Biol. 16, 1247–1255 (1996). 8622669 10.1128/mcb.16.3.1247 PMC231107

[b52] Lopez J. M. . Salt-resistant hypertension in mice lacking the guanylyl cyclase-A receptor for atrial natriuretic peptide. Nature 378, 65–68 (1995). 7477288 10.1038/378065a0

[b53] Nojiri T. . Atrial natriuretic peptide prevents cancer metastasis through vascular endothelial cells. Proc. Natl. Acad. Sci. USA 112, 4086–4091 (2015). 25775533 10.1073/pnas.1417273112 PMC4386325

[b54] Asano T. . Permanent genetic tagging of podocytes: fate of injured podocytes in a mouse model of glomerular sclerosis. J. Am. Soc. Nephrol. 16, 2257–2262 (2005). 15987751 10.1681/ASN.2004121134

[b55] Yokoi H. . Overexpression of connective tissue growth factor in podocytes worsens diabetic nephropathy in mice. Kidney Int. 73, 446–455 (2008). 18075496 10.1038/sj.ki.5002722

[b56] Saleem A. M. . A conditionally immortalized human podocyte cell line demonstrating nephrin and podocin expression. J. Am. Soc. Nephrol. 13, 630–638 (2002). 11856766 10.1681/ASN.V133630

[b57] Koga K. . MicroRNA-26a inhibits TGF-β -induced extracellular matrix protein expression in podocytes by targeting *CTGF* and is downregulated in diabetic nephropathy. Diabetologia 58, 2169–2180 (2015). 26063197 10.1007/s00125-015-3642-4

[b58] Yokoi H. . Pleiotrophin triggers inflammation and increased peritoneal permeability leading to peritoneal fibrosis. Kidney Int. 81, 160–169 (2012). 21881556 10.1038/ki.2011.305

