# Supplementary material for: Natriuretic peptide receptor guanylyl cyclase-A pathway counteracts glomerular injury evoked by aldosterone through p38 mitogen-activated protein kinase inhibition
Source: Sci Rep. 2017 Apr 21;7:46624. doi: 10.1038/srep46624 (PMC5399490; doi:10.1038/srep46624)
Supplement: Supplementary Figures [file srep46624-s1.pdf]

Natriuretic peptide receptor guanylyl cyclase-A pathway counteracts glomerular injury evoked by aldosterone through p38 mitogen-activated protein kinase inhibition

Yukiko Kato[1]; Kiyoshi Mori[2,3]; Masato Kasahara[4]; Keisuke Osaki[1]; Akira Ishii[1]; Keita P. Mori[1]; Naohiro Toda[1]; Shoko Ohno[1]; Takashige Kuwabara[1,5]; Takeshi Tokudome[6]; Ichiro Kishimoto[7]; Moin A. Saleem[8]; Taiji Matsusaka[9]; Kazuwa Nakao[10]; Masashi Mukoyama[1,5]; Motoko Yanagita[1]; Hideki Yokoi[1]

[1]Department of Nephrology, Graduate School of Medicine, Kyoto University, Kyoto, Japan, [2]School of Pharmaceutical Sciences, University of Shizuoka, Shizuoka, Japan, [3]Department of Nephrology and Kidney Research, Shizuoka General Hospital, Shizuoka, Japan, [4]Institute for Clinical and Translational Science, Nara Medical University Hospital, Kashihara, Japan, [5]Department of Nephrology, Kumamoto University Graduate School of Medical Sciences, Kumamoto, Japan, [6]Department of Biochemistry, National Cerebral and Cardiovascular Research Institute, Osaka, Japan, [7]Department of Endocrinology and Diabetes, Toyooka Public Hospital, Toyooka, Japan, [8]Academic Renal Unit, Bristol Children’s Hospital, University of Bristol, Bristol, UK, [9]Department of Molecular Life Sciences, Tokai University School of Medicine, Isehara, Japan, [10]Medical Innovation Center, Graduate School of Medicine, Kyoto University, Kyoto, Japan.

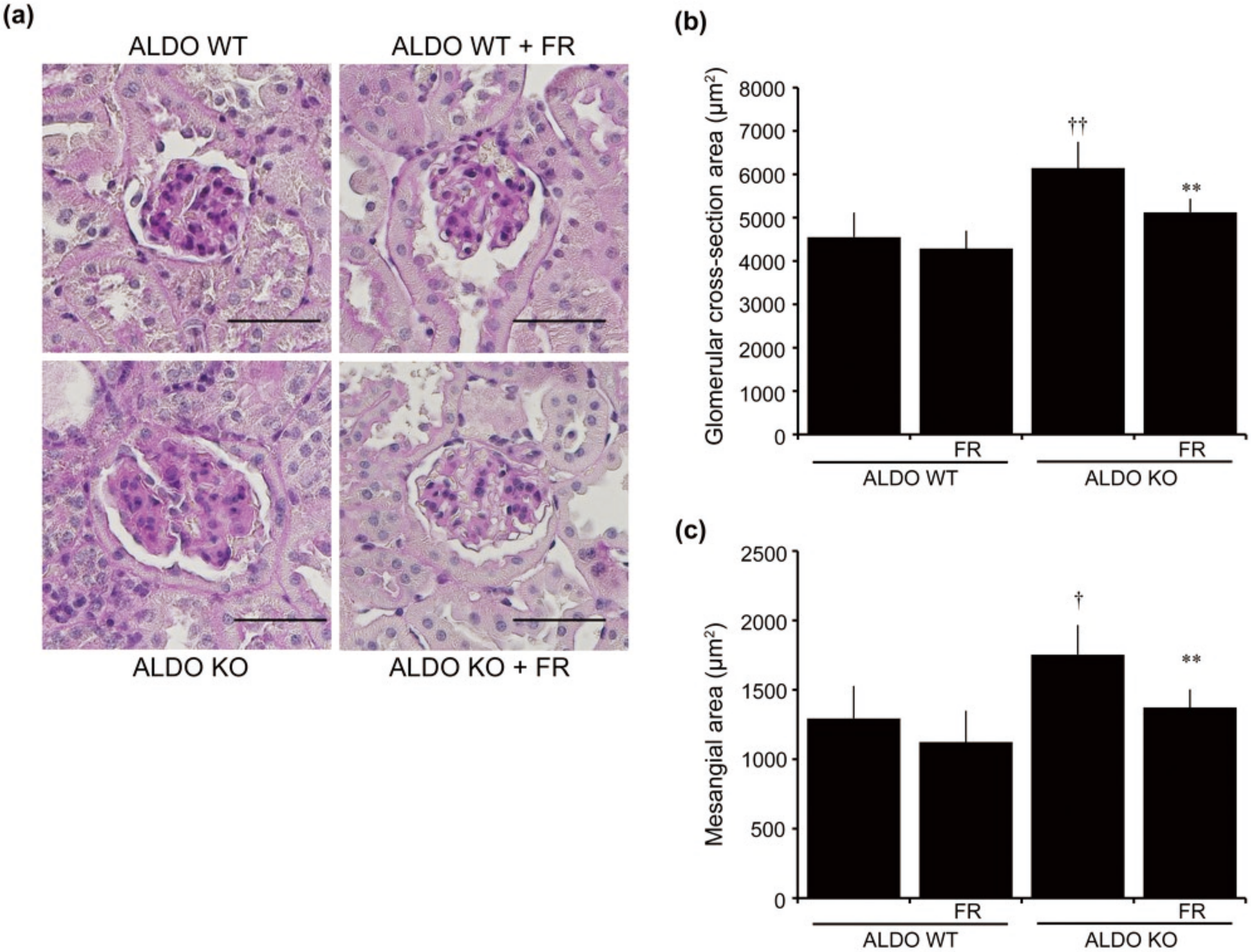

Supplementary Figure S1. Histological examination of superficial glomeruli in ALDO systemic GC-A KO mice. (**a**) Light microscopic analyses were performed at 4 weeks after aldosterone administration, stained with periodic acid-Schiff. In superficial glomeruli, ALDO systemic GC-A KO mice showed mild glomerular and mesangial hypertrophy. Treatment with FR167653 improved these changes. Scale bar, 50 μm. (**b**) Glomerular cross-sectional area and (**c**) mesangial area in superficial glomeruli at 4 weeks. Mean ± SEM. †$p < 0.05$, ††$p < 0.01$ vs. ALDO wild-type mice. *$p < 0.05$, **$p < 0.01$ vs. ALDOsystemic GC-A KO mice.

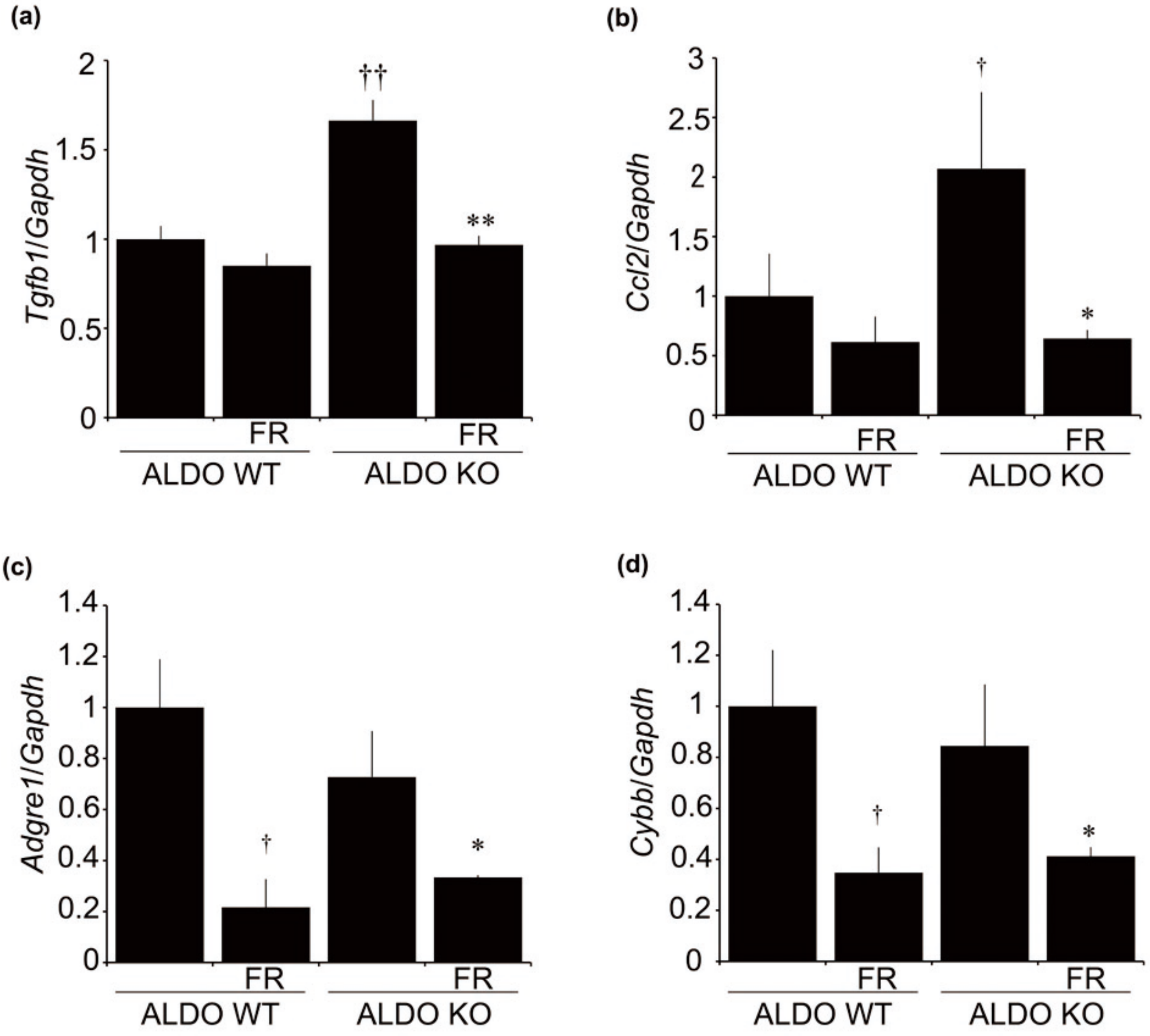

Supplementary Figure S2. Glomerular mRNA expression at 4 weeks after aldosterone administration. Real-time RT-PCR analysis of (**a**) *Tgfb1* (TGF-β1), (**b**) *Ccl2* (MCP1), (**c**) *Adgre1* (F4/80) and (**d**) *Cybb* (Cybb) are shown. n = 5, each. Mean ± SEM. *$p < 0.05$, **$p < 0.01$ vs. ALDO wild-type mice, †$p < 0.05$, ††$p < 0.01$, vs. ALDO systemic GC-A KO mice.

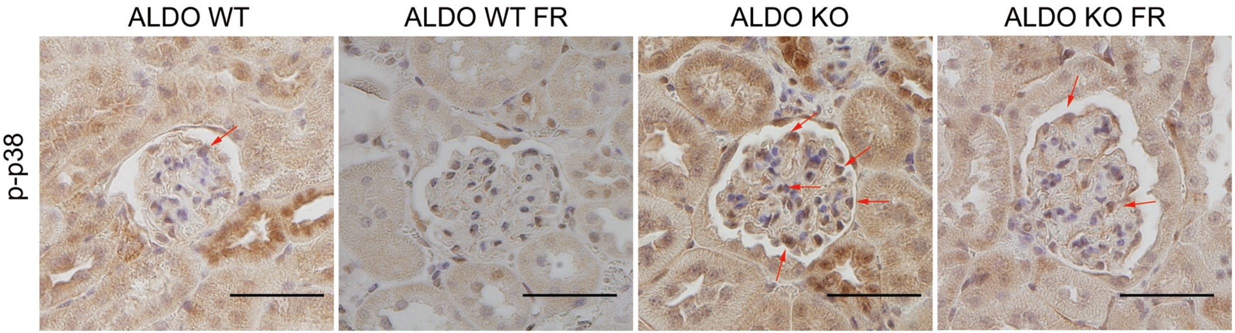

Supplementary Figure S3. Immunohistochemical study for phosphorylation of p38 MAPK in ALDO systemic GC-A KO mice with or without FR167653. Arrows, phopho-p38 MAPK-positive cells. Scale bar, 50 μm.

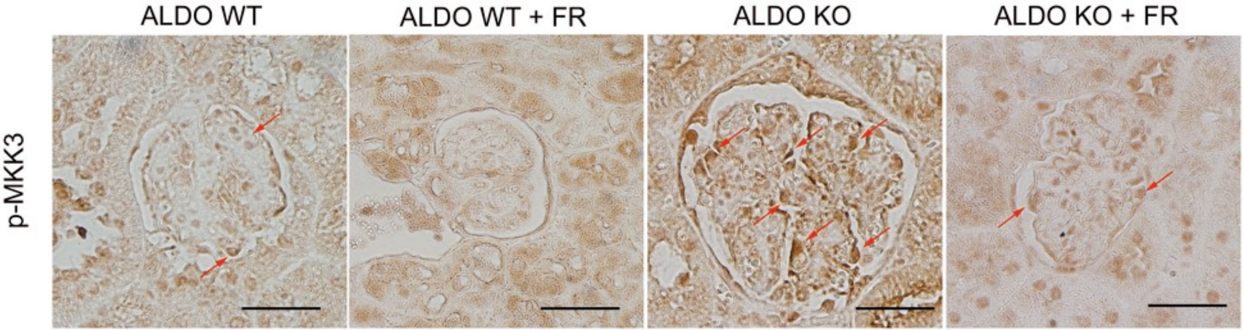

Supplementary Figure S4. Immunohistochemical study for phosphorylation of MKK3 in ALDO systemic GC-A KO mice with or without FR167653. Arrows, phopho-MKK3-positive cells. Scale bar, 50 µm.

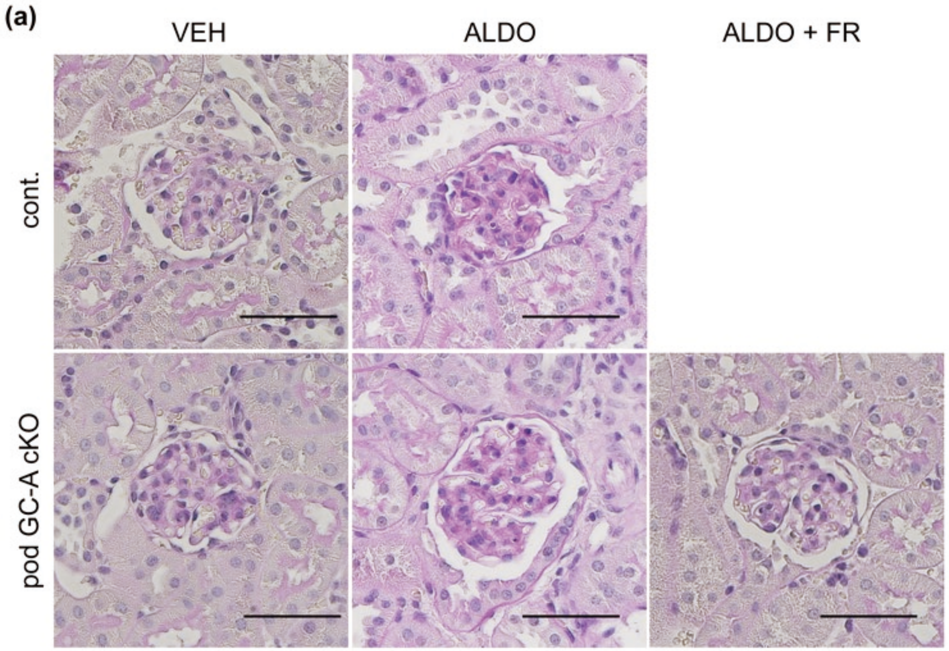

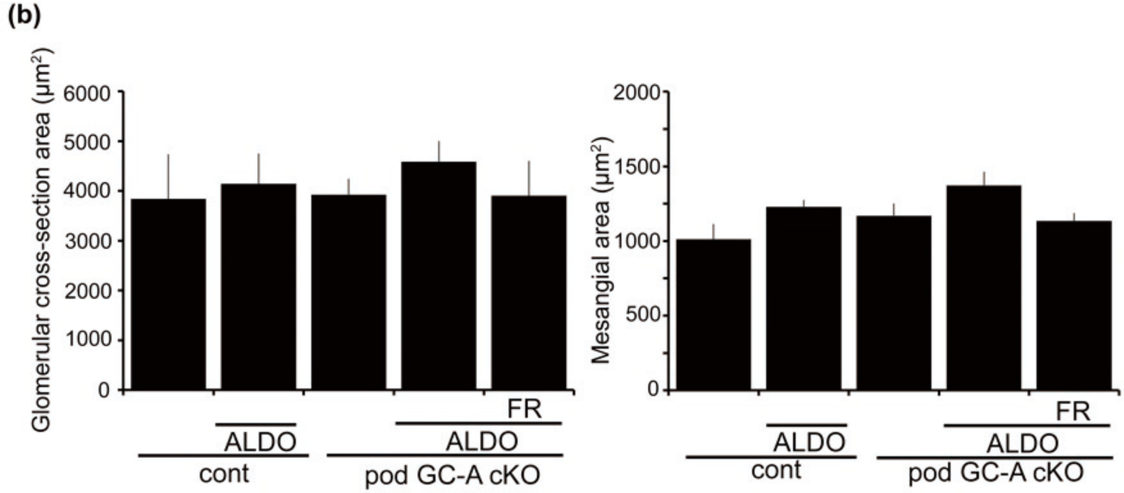

Supplementary Figure S5. (**a**) PAS staining of renal section of superficial glomeruli, and (**b**) their glomerular cross-sectional area and mesangial area in VEH or ALDO pod GC-A KO mice. FR167653 was administered to ALDO pod GC-A cKO mice. Scale bars, 50 μm. n = 5, each. Mean ± SEM.

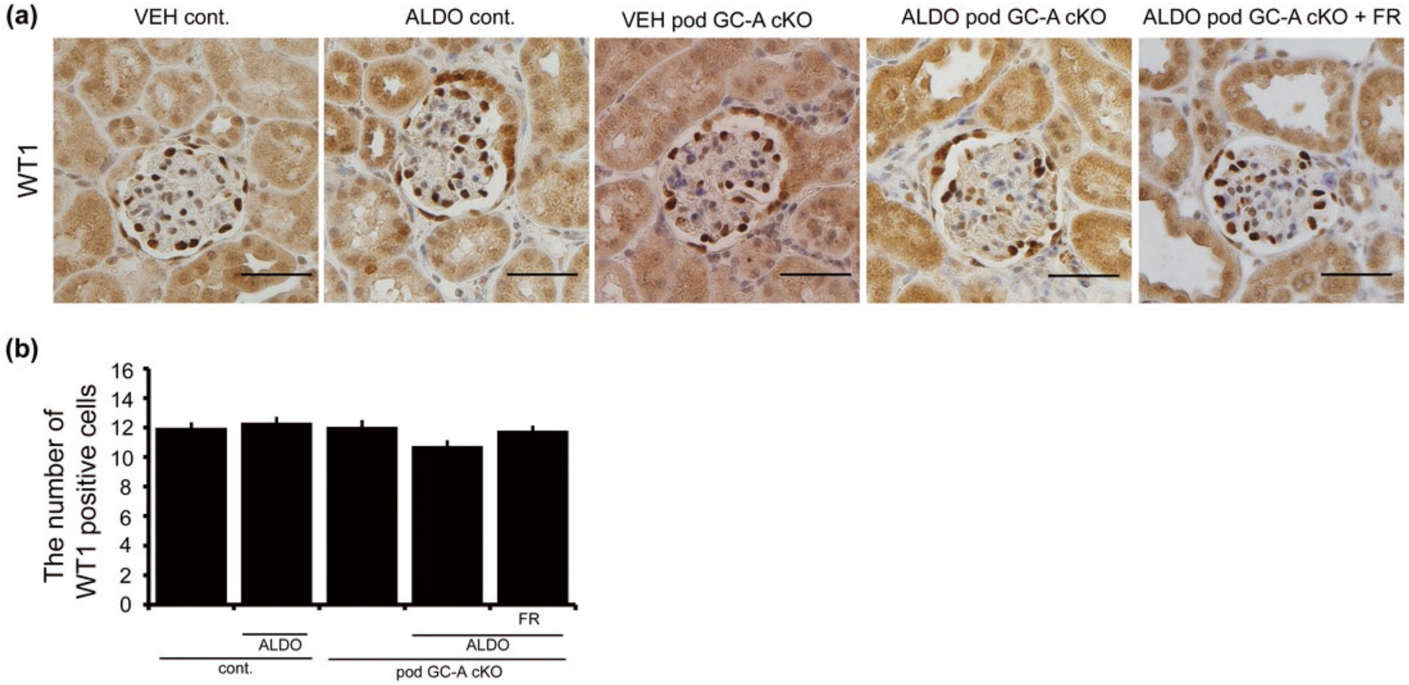

Supplementary Figure S6. (**a**) Immunohistochemical study for WT1 of superficial glomeruli in pod GC-A cKO mice. Administraion of aldosterone did not decrease WT1-positive cells in suferficial glomeruli. (**b**) The number of WT1 positive cells in superficial glmerulli. Mean ± SEM. Scale bar, 50 μm.
